# Preliminary Evaluation of a Granite Rock Dust Product for Pest Herbivore Management in Field Conditions

**DOI:** 10.3390/insects11120877

**Published:** 2020-12-11

**Authors:** Nicoletta Faraone, N. Kirk Hillier

**Affiliations:** 1 Chemistry Department, Acadia University, Wolfville, NS B4P 2R6, Canada; 2 Biology Department, Acadia University, Wolfville, NS B4P 2R6, Canada; kirk.hillier@acadiau.ca

**Keywords:** granite dust, repellent, silica-based product, ornamental plants, mineral formulation, herbivory

## Abstract

**Simple Summary:**

Rock dusts, including granite rock dust, are a rich source of silicon, one of the most abundant elements in the earth’s crust. Silicon exerts repellent, insecticidal and anti-ovipositional activities against agricultural pests. When accumulated on plant tissue, it can also form a mechanical barrier that increases resistance to pest attack. In the present work, we examined the effect of granite dust in managing herbivores under field conditions, evaluating the repellent and insecticidal activity against pests in lily, cabbage, and squash plants. Rock dust provided significant protection against specialist lily leaf beetles but was not effective in reducing general herbivore damage to field-grown cabbage and squash plants. Interestingly, rock dust also improved squash plant yield, increasing fruit size 2.5-fold. Overall, these results suggest granite as a beneficial alternative to synthetic pesticides with potential to manage pest herbivores, and to boost plant health.

**Abstract:**

The effects of granite rock dust in dry and aqueous formulations were evaluated under field conditions for control of insect pests in different crop systems and ornamental plants. We tested efficacy of crop protection following foliar applications on lily, squash, and cabbage plants by evaluating subsequent pest damage, overall plant health, and quantity of crops produced over one season. Lily plants treated with dry and aqueous formulations of rock dust were subject to lower herbivore damage (>1% and 11% herbivory damage, respectively) when compared to the controls (30% herbivory damage). Treatment on cabbage was less effective to protect plants against herbivory damage, and no statistically significant differences were reported within treatments. The foliar applications (dry and aqueous formulations) had positive impacts on growth of squash fruit resulting in a 2.5-fold increase in size relative to the control squash fruit. These results support the potential field application of granite dust to protect ornamental plants against herbivory attack, and reveal an alternative positive effect of the silica-based product on plant growth and development.

## 1. Introduction

The use of synthetic pesticides can have negative effects in the agricultural sector, causing environmental pollution and harm to humans and beneficial insects. Ecologically friendly pest management alternatives are in high demand for conventionally managed fields [1,2,3], as well as for ornamental plants [4,5]. Pesticides based on natural products (i.e., from plant origin and from natural sources) represent an important group of crop protectants that may be safer to humans and the environment with minimal residual effects [6,7]. Among them, mineral- and dust-based products (such as diatomaceous earth, kaolin clay, and silica formulations) have been widely used in crop protection, and are of increasing interest in the agricultural sector as crop pest management tools [8,9,10,11,12].

The use of silicon in agriculture as a pre-harvest treatment is beneficial as it does not leave residues on food or the environment, and it can be easily integrated with other pest management practices, including biological control strategies [13]. Several studies have shown a role for silicon-based products in enhancing resistance of plants to insect herbivores including folivores [14,15], phloem [16], cell content [17], and fruit feeders [18]. The protective effect of silica to plants against herbivores may be linked to its accumulation and polymerization in plant tissues, forming a mechanical barrier that increases resistance to pest attack [19,20,21]. Granite dust investigated in this study has similar effects and a similar mode of action as reported for other silica dust products, having repellent, insecticidal, and anti-ovipositional activities against Lepidoptera [15], two-spotted spider-mite (*Tetranychus urticae* Koch, Acari: Tetranychidae) [17], and multicoloured Asian lady beetle (*Harmonia axyridis* (Pallas), Coleoptera: Coccinellidae) [18]. The objective of this research was to evaluate the efficacy of this silica-based rock dust product under field conditions for managing insect pests in different horticultural systems. We tested the product on two crops and one ornamental plant variety. Lily plant (*Lilium* spp.) was selected as an important economically and aesthetically valuable horticultural commodity. In Canada, and particularly in the Atlantic Provinces, lily plants are heavily affected by invasive scarlet lily leaf beetles (*Lilioceris lilii* Scopoli, Coleoptera: Chrysomelidae) [22]. As the crop system, we selected cabbage plant (*Brassica oleracea* L.), mainly affected by diamondback moths (*Plutella xylostella* (L.), Lepidoptera: Plutellidae), flea beetles (*Phyllotreta* spp., Coleoptera: Chrysomelodae), and slugs. Finally, we evaluated the rock dust in a commercial acorn squash (*Cucurbita pepo* var. *turbinata* L.) field affected by striped cucumber beetles (*Acalymma vittatum* (F.), Coleoptera: Chrysomelidae) and squash bugs (*Anasa tristis* (De Geer), Hemiptera: Coreidae). These pests are particularly serious in the squash industry because they vector bacterial wilt [23] and the cucurbit yellow vine disease [24], respectively.

## 2. Materials and Methods

*Treatments*—Rock dust powder (mixture of granite dusts; particle size range 60–20 μm) [15,17] was provided by Heritage Memorials Ltd. (Windsor, NS, Canada). Efficacy of the dust for reducing pest damage was tested comparing two different product formulations: dry and in an aqueous solution (250 g/L). As positive control, a commercially available silica-based (i.e., diatomaceous earth) insecticidal product (Insectigone^®^, Woodstream Canada Corporation, Brampton, ON, Canada) was used in all the experimental trials (Table 1).

**Figure 1:**
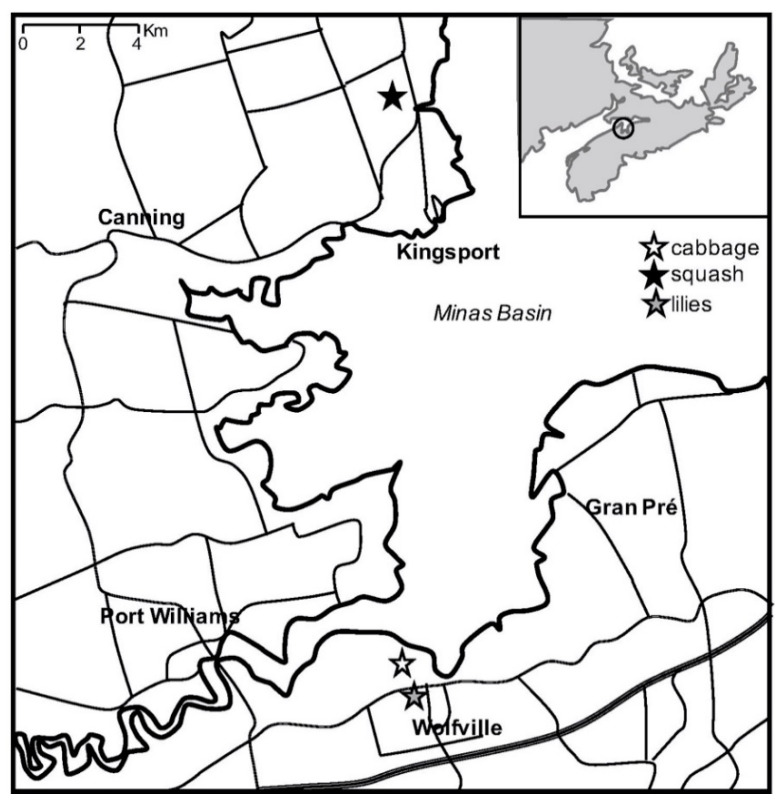
Overview of the location of three fields in Kings County (NS, Canada) (circled in the inset map).

*Field trials*—Field trials were performed during May–August 2018. Three different sites and plants/crops were selected (Figure 1), and the same experimental set up was used in all field trials. The aqueous formulation (250 g/L) was prepared by adding 250 g of dry dust to 1 L of water in a hand-sprayer, and shaken vigorously to blend the mixture well. Foliar spray applications were performed every 1–2 weeks, according to weather conditions (i.e., after a rainfall, wind under 20 km/h) and level of infestation. Because no studies have been carried out to determine the potential impact on beneficial insects to date, plants were treated early in the morning to minimize potential of direct exposure to pollinators. During application, the nozzle was held 20–30 cm from the plant and sprayed until to the point of run-off. For dry formulations, treatments were applied using a cup (500 mL volume) covered with a fine mosquito net, and dry dust was sprinkled in order to provide uniform coverage of the plant surface. The estimated volume of liquid formulation applied per plant/application was approximately 100–150 mL (25–37.5 g of dry material), and about 80–100 g of material per plant/application for dry dust treatments.

*Insects*—Insect pests commonly observed and recorded on the lily field site were scarlet lily leaf beetles (*L. lilii*). In the cabbage field, diamondback moths (*P. xylostella*), flea beetles (*Phyllotreta* spp.), and slugs were mainly responsible of the detected and recorded herbivory damage. Presence of striped cucumber beetles (*A. vittatum*) and squash bugs (*A. tristis*) was recorded in the acorn squash field site and linked to pest damage.

*Lily plot*—*Lilium* spp. “Asiatic hybrid lily” (purchased from Vesey’s Seeds Ltd., York, PE, Canada) was used for lily field trials. Bulbs were overwintered at Nelson Garden (private property located in Falmouth, NS, Canada) from December 2017. Bulbs were (plot not previously subjected to synthetic pesticide exposure) located at the K.C. Irving Environmental Science Centre (45°5′15″ N 64°22′5″ W), Acadia University (Wolfville, NS, Canada). The experimental plot (3.66 × 7.32 m) was divided into 12 blocks (3 columns × 4 rows; 1.1 × 1.1 m). Bulbs were planted in soil at a depth of 5–10 cm in April 2018, and treatments were performed after a month, when plants had reached 20–30 cm high. Manual weed removal was carried out weekly. Treatments were arranged in a randomized complete block design with three replicates of each treatment per block (containing three plants each, *n* = 9). Damage caused by caused by lily leaf beetles (*L. lilii*) was evaluated weekly counting the number of feeding holes present on each leaf (0.2–0.5 cm in diameter). Each lily plant had an average of 28 leaves (calculated based on the number of leaves recorded for each plant); we scored 0 when no damage was detected and 15 for a leaf that reported severe damage and was almost detached, and we assigned a score of 420 (28 × 15) for a damage equal to 100% (a plant with severe damage). Values were converted into percentages. In September 2018, lily plants were gently removed from the experimental plot and washed with water to remove soil and treatment residuals to assess plant mass, as well as stem, root, and leaf size [25]. From each plant, total number of leaves were counted, and five bottom leaves were measured (length and width), sampled and stored at −20 °C in plastic bags for further analyses, while the other plant material (leaves, stem, and roots) was transferred into paper bags and oven-dried at 70 °C for 48 h. The dry weight of each plant was recorded.

*Cabbage plot*—Cabbage plants (*B. oleracea* L.) were grown in the K.C. Irving Centre greenhouse (18 ± 2 °C, 16:8 L:D, 65 ± 5% R.H.) at Acadia University, Wolfville (NS, Canada) in 100 mm diameter pots containing Pro-Mix potting soil, and watered as needed. At four to six weeks of growth, plants were transferred in June 2018 to an external plot (not previously subjected to synthetic pesticide exposure) located at the Acadia University Community Garden (45°5′43″ N 64°22′8″ W; Wolfville, NS, Canada). The experimental plot (4 × 6 m) was divided into blocks (1.1 × 1.1 m) for a total of 12 blocks (3 columns × 4 rows). Cabbage transplants were planted into soil at a depth of 2–5 cm, and treatments were applied after 1 day. Treatments were arranged in a randomized complete block design with three replicates of each treatment per block (containing three plants each, *n* = 9). Every week, damage caused by diamondback moths (*P. xylostella*), flea beetles (*Phyllotreta* spp.), and slugs was visually evaluated and reported as a percentage. Cabbage plant was visually divided into 4 sections (25% of the plant) and each section was assessed for leaf loss and herbivore damage. Manual weed removal was carried out weekly. In September 2018, cabbage plants were gently removed from the experimental plot and washed with water to remove soil and treatment residuals to assess plant mass, stem, root, leaf size (measuring leaf width and length), and number. From each plant, five bottom leaves were sampled and stored at −20 °C in plastic bags for further analyses, while the other plant material was transferred into paper bags and oven-dried at 70 °C for 48 h. The dry weight of each plant was recorded.

**Figure 2:**
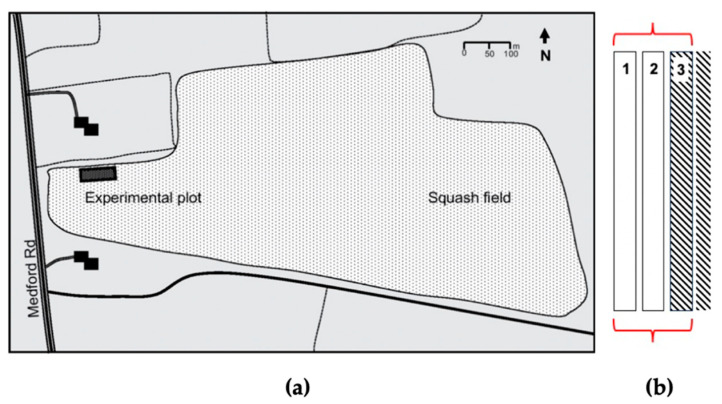
(**a**) Overview of buttercup squash field in Canning (NS, Canada). (**b**) Section of the rows: columns 1 and 2 were part of the trial, while column 3 (dashed lines, confining with the pesticide treated section) was considered as the buffer line and not included in the analysis.

*Squash plot*—Acorn squash seeds (*C. pepo*) were planted the first week of June 2018 by machine in rows 1.5–2.0 m apart, with an in-row spacing of 35–46 cm. One section of the squash field, located in Canning (45°10′20″ N 64°21′42″ W; NS, Canada), was selected as the experimental plot (50 × 3 m, located on the north-west corner adjacent to the border) (Figure 2) and not subjected to conventional insecticide and herbicide spraying. The rest of the field was treated twice with Admire^®^ (Bayer, Mississauga, ON, Canada) and Poast^®^ Ultra (BASF, Saint-Léonard, QC, Canada). The plot was organized into blocks (5 × 1 m) for a total of 30 blocks (3 columns × 10 rows). The column adjacent to the pesticide treated section was considered as buffer line and not included in the analysis because of possible drift during pesticide application. There were three to four replicates of each treatment (containing a maximum of 7 plants each), arranged in a randomized complete block design. Every week, pest damage was visually evaluated following the same approach described for cabbage plants, and reported as a percentage. Manual weed removal was carried out weekly. In September 2018, squash fruits were collected from the experimental plot, washed with water to remove soil and treatment residuals to assess fresh weight and diameter. Squash material was chopped and transferred into paper bags and oven-dried at 70 °C for 48–72 h. The dry weight of each squash was then recorded. From each squash, seeds were collected and air-dried at room temperature for about 2 weeks, and the dry weight of 10 seeds was recorded. Sampled seeds were then stored at −20 °C in plastic bags for further elemental analyses [26].

*Statistical analysis*—Statistical analyses were conducted with RStudio Version 01.1453 [27]. Data with non-normal distribution were subjected to nonparametric tests. The overall evaluation of treatment effect on herbivory attack over time was performed using the repeated measure approach with linear mixed-effect model (lmer, package nmle). Time and treatments were defined as fixed factors, while plant was the random effect nested within the block (plot) [28]. Damage over time on each lily plant was square-root transformed. The F test (sum of squares) with Kenward–Roger approximation [29] was performed on the model to evaluate the best fit, and a post-hoc test (least square means) for multiple comparisons (using lmerTest package) was performed to determine differences between groups [30]. Assessment of damage (as well as other parameters such as dry mass, root and stem length, leaf size, etc.) at the end of the experiment was performed by using only data from the last week of observation. Data on lily plant damage, lily leaf length and width, and dry weight were sqrt-transformed. Data with normal distribution were analyzed by ANOVA followed by, in case of significant effects, a post-hoc pairwise comparison (*t*-test). Non-normal data were analyzed through a Kruskal–Wallis test, followed by a post-hoc analysis with the Dunn test (dunnTest function in the FSA package). Differences were considered significant at *p* ≤ 0.05. 

## 3. Results

### 3.1. Lily Plot 

**Figure 3:**
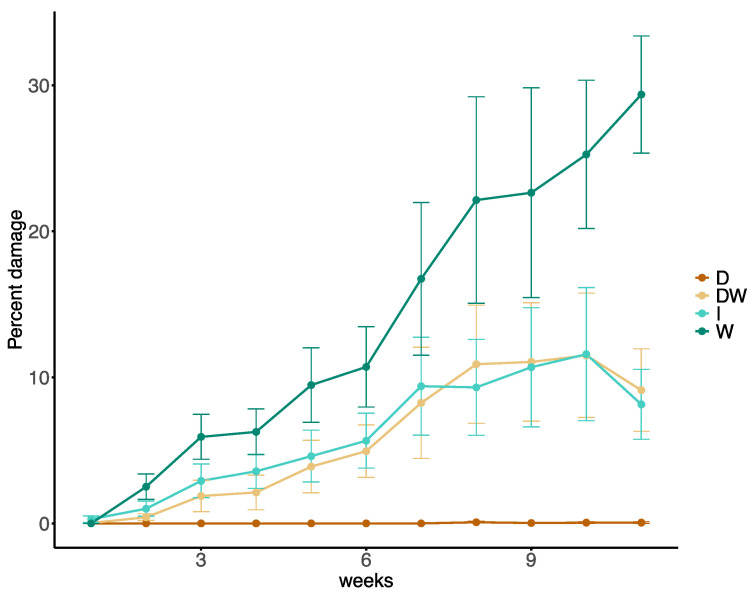
Percentage of herbivory damage (±SEM) caused by lily leaf beetles (*L. lilii*) at different time points on lily plants at different treatments (W = control; D = dry dust; DW = dust with water; I = Insectigone^®^).

Rock dust treatment was effective in reducing herbivore damage to lily plants. Among treatments, dry dust application was able to reduce herbivore damage (F_3,32_ = 11.36, *p* < 0.001) and it provided constant protection over time (F_10,350_ = 27.59, *p* < 0.001), with nearly 0% leaf damage observed over the 11-week experimental period. Moreover, overall herbivore damage in control plots significantly increased over time (Figure 3). 

At the end of the trial, dry rock dust treated plants had significantly less damage (*p* < 0.001), followed by dust mixed with water (*p* < 0.01) and Insectigone^®^ treatments (*p* < 0.01) which had 12% herbivory damage. All treatments were significantly more effective than control (χ^2^ = 25.04, df = 3, *p* < 0.001), which had up to 30% of herbivory damage after 11 weeks (Table 2).

The relative impact of rock dust treatments on plant parameters did not significantly differ from the other treatments. Dry weight of lily plants was the same in all treatments (χ^2^ = 2.48, df = 3, *p* = 0.478), as was root length (χ^2^ = 1.79, df = 3, *p* = 0.617) and total number of leaves collected from each plant (F_3,32_ = 0.43, *p* = 0.75). Lily leaf length (χ^2^ = 5.61, df = 3, *p* = 0.132) and width (χ^2^ = 5.27, df = 3, *p* = 0.152) were not affected by any of the treatments.

### 3.2. Cabbage Plot

The application of rock dust on cabbage did not provide significantly reduce herbivory relative to control (F_3,32_ = 0.405, *p* = 0.750), and herbivore damage increased over time (F_8,280_ = 23.57, *p* < 0.001), causing a negative impact on plant health. Although Insectigone^®^ treated cabbage plants experienced high damage (mostly caused by wild animals, such as deer and racoons), their dry mass was significantly greater (χ^2^ = 17.78, df = 3, *p* < 0.001) than those treated with control and dust treatments (Table 3). 

Treatments had an impact on leaf length (F_3,176_ = 3.92, *p* = 0.01) and width (χ^2^ = 12.4, df = 3, *p* < 0.01). Cabbage leaves treated with rock dust in aqueous formulation were smaller in width (*p* = 0.02) and length (*p* = 0.01) compared to the other treatments (Table 4).

### 3.3. Squash Plot

Treatments had a significant impact on fruit weight (χ^2^ = 14.47, df = 3, *p* > 0.01). Insectigone^®^ and dry dust treatments caused a 2.5-fold increase in fruit weight (Table 5) than the control fruits. However, none of the treatments were effective in reducing herbivore damage (F_3,7_ = 2.146, *p* = 0.1783), which remained constant over the duration of the experiment (F_7,70_ = 0.269, *p* = 0.964). No significant difference was observed between seed weights of different treatments (χ^2^ = 4.51, df = 3, *p* = 0.212).

## 4. Discussion

Application of rock dust had variable effects in different plant systems, and differing efficacy in terms of reducing herbivore damage. The product was particularly effective in reducing damage from scarlet lily beetle on lily plants. However, a similar efficacy was not observed for cabbage and squash plants, which experienced significant foliar damage. Reduced herbivory has been previously demonstrated by rock dust under lab conditions, where different formulations in dry and aqueous formats exerted a repellent and insecticidal action towards lepidopteran [15] and coleopteran pests [18]. Moreover, it was observed that the plants treated with granite dust through soil application were subsequently repellent and acaricidal for two-spotted spider mites (*Tetranychus urticae*) [17]. 

Elemental analysis previously performed on samples of rock dust [17] showed a high content of silicon present in the form of SiO_2_ (around 60% of the overall composition). This further suggests a correlation with silicon as an active ingredient for pest control as previously reported for other similar organic origin materials based on silica dust (such as diatomaceous earth). Such products were particularly effective in controlling storage pests [31,32], and exert a similar effect and mode of action to the rock dust in protecting plants against abiotic and biotic factors [16,19,20,33,34]. The action of Si in promoting plant resistance has not been well characterized, but evidence from studies on plant metabolic processes support multiple combined effects rather than one single effect [13], with silicon acting either directly or indirectly to inhibit insect herbivory [35,36]. In our previous study, the accumulation of silicon on the leaf surface following foliar application of rock dust was positively correlated to the repellency and acaricidal action reported toward two-spotted spider mites [17]. In addition to being susceptible to rock dust by direct contact, changes were noted in the structure of treated leaves (mineral accumulation and epidermal thickening) which are proposed to have indirectly impacted the feeding ability of mites [17].

Testing novel bioproducts under field conditions requires consideration of multiple variables, such as weather, humidity, and different herbivores that may impact insecticidal performance [37,38,39]; moreover, the selection of an appropriate formulation that can improve product stability and viability may reduce inconsistency of lab and field performances [40]. In this study, variable results reported for rock dust efficacy under lab and field conditions highlight the need to optimize formulation of the product and delivery [41] for specific target pests/plants. Although under lab conditions granite rock dust proved to be effective in controlling lepidopteran pests (such as diamondback moth and cabbage looper) [15], the application under field conditions did not exert the expected repellent and insecticidal activity on cabbage plants. Cabbage plants were subjected to high damage (>90%) caused not only by insect herbivores; similarly, squash plants were damaged extensively by striped cucumber beetles and squash bugs [23,24].

The rock dust treatment had an impact on squash fruit production. The size of squash fruits produced by plants treated with rock dust was significantly greater (i.e., 2.5-fold increase in mass) than those from the control plants. A similar trend was observed for the diatomaceous earth treatment, producing squash fruits with similar weights as those treated with rock dust. Foliar application of rock dust material has beneficial effects on plant productivity which can be translated into a more efficient crop yield with increased Si uptake [17,42,43,44]. The application of mineral dust, not just through foliar application but also as soil amendment, may provide important nutrients and elements for plant growth, and it has been reported to have a potential impact on amount of crop grown [44,45]. Particularly, silicon has been linked to increased crop productivity, and was very effective in increasing production and herbivore resistance in rice [46] and sugarcane [47], as well as plant growth and fruit quality in strawberry [48] and tomatoes [49,50]. Lily leaves treated with diatomaceous earth-based product were significantly larger, and squash plants treated with granite dust produced squash fruits with sizes significantly greater than those from the control plants. An opposite trend was observed for cabbage plants—leaves treated with rock dust in aqueous formulation and Insectigone^®^ were smaller. This may be a result of variable silicon uptake among plant species [51].

Additional studies need to be carried out under field conditions in order to confirm the efficacy of the granite dust, to determine crops that will most benefit by application, and to optimize delivery. In terms of a specific mode of action, it remains unclear how granite dust, which causes a reduction in insect performance and plant damage, affects herbivores and if it is responsible in mediating defense and plant productivity through Si uptake. Overall, this novel rock dust material presents promising results with regard to being employed as a pest repellent product and potentially as a soil amendment for promoting crop growth. 

## 5. Conclusions

The application of rock dust under field conditions had variable results in controlling herbivores. It was highly effective in managing lily beetles on lily plants, but it did not protect cabbage and squash plants from lepidopteran and coleopteran pests, respectively. The foliar application of rock dust was also positively correlated with the increase in squash size and weight. Optimal pest control was not equivalent among studied plants; however, these findings reflect variation in efficacy related to individual plant species, and their natural pest complexes. Additionally, high Si content present in the rock dust is proposed to have exerted beneficial effects for growth of lilies and squash when applied as a foliar treatment. Overall, granite rock dust has potential as a valuable pest management tool in agricultural and ornamental settings.

## Figures and Tables

**Table 1:** List of treatments used in the field trials.

Treatment	ID	Description
Water	W	Control
Dust	D	Dry dust powder, mixture of granite dusts
Dust with water	DW	Dry dust powder mixed with water at 250 g/L
Insectigone^®^	I	Diatomaceous earth-based insecticidal dry powder

**Table 2:** Mean percent herbivory damage (±SEM) caused by caused by lily leaf beetles (*L. lilii*) on lily plants for different treatments of rock dusts and diatomaceous earth. Data collected at the end of the trial (*n* = 9).

Treatment	Herbivory Damage % (±SEM) ^‡^		
		Z	*p*
W	31.59 (±2.71)	-	-
D	0.05 (±0.05)	−4.94	**<0.0001**
DW	11.51 (±4.26)	−1.95	**0.04**
I	11.59 (±4.56)	−1.94	**0.03**

^‡^ Dunn test. Bold values indicate significant differences of means to the control.

**Table 3:** Mean percent (±SEM) herbivory damage caused by diamondback moths (*P. xylostella*), flea beetles (*Phyllotreta* spp.) and slugs, and dry mass on cabbage plants for different treatments of rock dusts and diatomaceous earth. Data collected at the end of the trial (*n* = 9).

Treatment	Dry Mass (g) (±SEM) ^‡^		
		Z	*p*
W	46.67 (±8.19)	-	-
D	76.82 (±16.59)	−1.58	0.07
DW	45.71 (±5.77)	0.268	0.394
I	138.90 (±23.47)	−3.468	**<0.001**

^‡^ Dunn test. Bold values indicate significant differences of means to the control.

**Table 4:** Mean percent (±SEM) length and width of cabbage leaves for different treatments of rock dusts and diatomaceous earth. Data collected at the end of the trial (*n* = 45).

Treatment	Leaf Width (cm) (±SEM) ^‡^			Leaf Length (cm) (±SEM) *		
		Z	*p*		t	*p*
W	13.77 (±0.60)	-	-	19.58 (±0.71)	-	-
D	14.58 (±0.40)	−1.031	0.227	18.84 (±0.50)	−0.893	0.746
DW	12.23 (±0.45)	2.251	**0.02**	17.02 (±0.57)	−3.146	**0.012**
I	14.05 (±0.43)	−0.483	0.315	17.74 (±0.47)	−2.253	0.127

^‡^ Dunn test. * Pairwise *t*-test. Bold values indicate significant differences of means to the control.

**Table 5:** Mean percent (±SEM) dry mass on squash fruits for different treatments of rock dusts and diatomaceous earth. Data collected at the end of the trial (*n* = 11).

Treatment	Dry Mass (g) (±SEM) ^‡^		
		Z	*p*
W	159.55 (±28.64)	-	-
D	406.58 (±25.09)	−2.967	**<0.01**
DW	302.38 (±39.20)	−1.815	0.05
I	437.06 (±49.15)	−3.458	**0.001**

^‡^ Dunn test. Bold values indicate significant differences of means to the control.
